# Geographic Variation in Floral Color and Reflectance Correlates With Temperature and Colonization History

**DOI:** 10.3389/fpls.2020.00991

**Published:** 2020-06-30

**Authors:** Matthew H. Koski, Laura F. Galloway

**Affiliations:** ^1^ Department of Biological Sciences, Clemson University, Clemson, SC, United States; ^2^ Department of Biology, University of Virginia, Charlottesville, VA, United States

**Keywords:** biogeography, genetic drift, flower color, insect vision, range expansion, thermoregulation

## Abstract

Petal color variation within species is common and may be molded by abiotic or biotic selection pressures, or neutral population structure. For example, darker flowers may be favored in cooler environments because they absorb more solar radiation, elevating the temperature of reproductive structures. Additionally, flower color may evolve to attract the dominant or most efficient pollinator type in a given population. Here, we evaluate geographic variation in petal coloration across the range of *Campanula americana* in Eastern North America and test whether color covaries with abiotic factors, the pollination community, and genetic structure established through post-glacial expansion. Consistent with other studies, flowers from cooler, higher latitude populations were less reflective across the UV-NIR spectrum than those from warmer populations. Local temperature explained variation in petal reflectance better than the pollinator community or colonization history. Petal color perceived by trichromatic bee pollinators displayed a strong longitudinal pattern but was unassociated with climatic factors and the pollinator community. Instead, pollinator-perceived color was tightly correlated with the geographic distance from *C. americana*'s glacial refugium. In total, abiotic conditions appear to shape large-scale geographic variation in the intensity of petal reflectance while genetic structure is the strongest driver of pollinator-perceived petal coloration. This study highlights the importance of abiotic factors and historical processes associated with range expansion as major evolutionary forces shaping diversity of flower coloration on large geographic scales.

## Introduction

Floral coloration can have strong effects on plant-pollinator interactions (e.g., Gigord et al., 2001; Guitián et al., 2017), and recent evidence highlights its impact on the microclimate experienced by pollen and ovules (Lacey et al., 2010; Koski and Ashman, 2015; van der Kooi et al., 2019). Correlating geographic variation in petal coloration with ecological parameters such as pollinator communities or climatic conditions is one approach that can elucidate the degree to which putative selective agents may shape the evolution of color across large spatial scales (Brunet, 2009; Sobral et al., 2015). However, neutral population genetic structure has the potential to contribute to geographic variation in petal color as well (Rausher, 2008). For instance, genetic structuring due to range expansion and contraction have been posited to shape geographic petal color variation in *Gentiana lutea* (Sobral et al., 2015). An understanding of both adaptive and non-adaptive processes can be crucial for explaining floral color evolution (Streisfeld and Kohn, 2005; Streisfeld and Kohn, 2007; Hopkins et al., 2012; Baranzelli et al., 2014; Berardi et al., 2016).

Pollinators select on flower color attributes such as brightness and hue (Caruso et al., 2010; Sletvold et al., 2016), so the most commonly tested driver of geographic variation in floral coloration is the pollination community. Pollinator guilds often differ in their sensory biases for flower color (Schiestl and Johnson, 2013) and if pollinator communities differ between populations they can drive geographically divergent selection. There is strong evidence that pollinators contribute to flower color disparities among populations of the same species in some systems (Streisfeld and Kohn, 2007; Sobral et al., 2015; Streinzer et al., 2019). On the other hand, differences in pollinator communities are insufficient for explaining color variation in a number of others (Schemske and Bierzychudek, 2007; Thairu and Brunet, 2015). Thus, non-pollinator agents of selection are often invoked to shape variation in flower coloration (e.g., Strauss and Whittall, 2006).

Abiotic conditions such as temperature (Coberly and Rausher, 2003; Lacey et al., 2010; Koski and Galloway, 2018), water availability (Warren and Mackenzie, 2001; Arista et al., 2013; Vaidya et al., 2018), or solar radiation (Arista et al., 2013; Koski and Ashman, 2015), can act as selective agents on flower color either directly or indirectly. As a result, large scale patterns of flower color have been linked with climatic gradients (Arista et al., 2013; Koski and Ashman, 2015; Koski and Galloway, 2018). For example, less reflective (darker) flowers have the ability to warm reproductive structures more efficiently than lighter flowers through absorption of more solar radiation, which can increase reproductive success in cooler climates (Lacey et al., 2010). Pollinators have also been shown to prefer warmer flowers in cooler environments (van der Kooi et al., 2019), potentially providing an advantage to darker flowers. Darker coloration can also be favored in drought conditions (Warren and Mackenzie, 2001). For example, in *Boechera stricta*, there is a greater likelihood of pigmented flowers in low elevation populations which may be due to elevated drought tolerance of pigmented morphs (Vaidya et al., 2018). Models that incorporate the effects of both abiotic and pollinator attributes of the environment on coloration can help to parse the impacts of each on floral color.

It is also important to consider the effects of population genetic structure when examining large-scale geographic covariance between coloration and ecological factors (Rausher, 2008). Numerous studies have found natural selection is stronger than drift in driving among-population floral color variation (Schemske and Bierzychudek, 2007; Streisfeld and Kohn, 2007; Jorgensen et al., 2006; Hopkins et al., 2012). However, to date very few studies have examined the effects of population structure imposed by historical colonization on petal color variation across the range of a species (however see Baranzelli et al., 2014). During range expansion, small founder populations that establish beyond a range edge are most likely derived from range-edge populations (Hallatschek and Nelson, 2008). Serial genetic bottlenecks experienced through founder events should establish geographic population structure with genetic diversity declining with increasing distance from glacial refugia (e.g., Willi et al., 2018; Koski et al., 2019). Floral color could show spatial patterns consistent with neutral evolution along expansion routes. In this scenario, geospatial variation in color would be a signature of historical processes rather than geographically divergent natural selection, though both scenarios are not mutually exclusive.

*Campanula americana* is a widespread plant in Eastern North America with petals that range from blue to violet. It is pollinated by bumblebees which are very effective at pollen transfer, and small solitary bees that are relatively ineffective pollinators (Koski et al., 2018). It displays variation in pollen coloration that is genetically uncorrelated with petal color (Koski and Galloway, 2018; Koski et al., 2020). Geographic patterns of petal coloration have not been evaluated. Here, we correlate range-wide variation in signatures of petal reflectance with data on pollinator communities, climatic variables, and post-glacial expansion for 24 populations of *C. americana*. We address the following questions: 1) Does petal coloration display geographic variation? and 2) If so, is it driven by pollinator communities, climatic variables, or historical range expansion? If selection is important in shaping color variation, we predict that a) petals will be less reflective in more northern populations where elevated absorbance is likely favored in cool environments, and b) petal color as perceived by pollinators will be correlated with the relative abundances of effective bumblebee pollinators and ineffective small solitary bees. Alternatively, if genetic structure imposed by post-glacial colonization is more important in structuring color variation than contemporary selection, we predict that the distance from the glacial refugium will strongly predict all aspects of petal coloration.

## Methods

### System

*Campanula americana* (=*Campanulastrum americanum*, Campanulaceae) is a bee-pollinated annual to biennial species of Eastern North America that flowers from late June to early September. It occurs frequently at forest edges and less commonly in the understory and spans a large environmental gradient from Florida to Minnesota. It is largely outcrossing (Galloway et al., 2003; Koski et al., 2019) and is visited by a variety of bee species with varying pollination effectiveness. Bumblebees are highly effective at pollen transfer and affect higher seed set per flower visit than smaller solitary bees (*Megachile campanulae* (Megachilidae); various Halictidae; Lau and Galloway, 2004; Koski et al., 2018). Across its range west of the Appalachian Mountains, it is pollen limited on average (mean for 24 populations= 19%; Koski et al., 2017). However, populations that experience higher bumblebee visitation rates are less pollen limited (Koski et al., 2017; Koski et al., 2018). The color of pollen varies from white to deep purple (Lau and Galloway, 2004) and darker pollen has elevated germination potential under heat stress (Koski and Galloway, 2018). Bumblebees learn to associate rewards with pollen color, and in natural populations, *M. campanulae* prefers flowers with darker pollen (Ison et al., 2019). Thus, color variation has the ability to impact pollinator choice. Pollen color and petal color are not genetically correlated (Koski et al., 2020).

In this study we focus on 24 populations west of the Appalachian Mountains which form a clade that is divergent from populations in the Appalachians, as well as those east of the Appalachians (Barnard-Kubow et al., 2015). Population genomic analyses support an origin for this Western clade in the Appalachian Plateau of Southwestern Kentucky (Koski et al., 2019). This location served as the most-likely region of glacial refugia during the Pleistocene glaciation with stepwise colonization westward and northward following glacial recession. Further genomic analyses support genetic clustering west and east of the Mississippi River (Prior et al., in revision).

### Plant Material and Petal Reflectance Measurements

Ripe fruits were field collected from the 24 C*. americana* populations in Summer 2015. We sowed seed from 25 maternal families from each population in growth chambers at the University of Virginia. A single seedling from each family was vernalized and then transplanted into the greenhouse (see Koski et al., 2017 for detail on plant propagation). Upon flowering, we collected a single first day flower from an average of 15.2 plants per population (n=365 plants). Using an Ocean Optics spectrometer with a UV-Vis light source, we measured petal reflectance with a reflection probe held at a 90 angle. Since petals are not glossy, specular reflection was not likely to contribute strongly to spectral outputs. Within a given plant, flowers are similar in coloration (M. Koski, pers. obs.).

**Figure 1:**
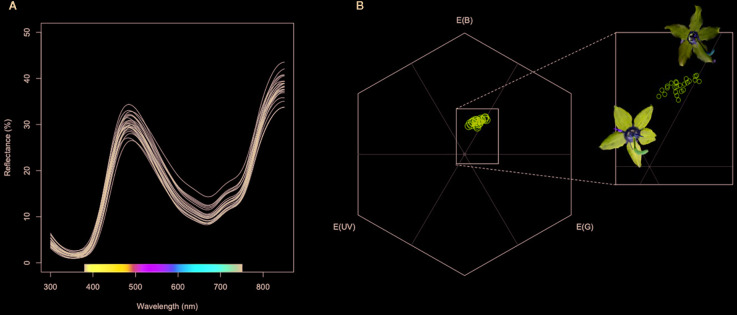
**(A)** Spectral reflectance of petals of *Campanula americana*. Each curve is the average spectral reflectance for a single population (N=24). **(B)** Mean spectral reflectance for each population placed in hexagonal color space of the trichromatic pollinator *Bombus impatiens*. The inset focuses on the blue and blue-green sectors of color space in which *C. americana* petals fall. Flower images display petals with a low *x* and low *y* coordinate (deeper purple) and petals with higher *x* and *y* coordinates (lighter purple).

*Campanula americana* petals display a peak reflectance in the blue range around 430nm, reduced reflectance between 500 and 650nm and an increase in reflectance in the NIR range (**Figure 1**). UV reflectance from petals is low in these populations west of the Appalachians. We first scored reflectance metrics that captured variation in reflectance curves but did not take into consideration pollinator visual systems. These included Mean Reflectance (i.e., mean brightness), Peak Blue Reflectance, and Peak NIR reflectance. Mean reflectance is the average % reflectance from 300-850 nm. Peak blue reflectance is the highest % reflectance value recorded between 410 and 500 nm, and NIR reflectance is the % reflectance recorded at 800 nm. Similar metrics have been used as estimates for the potential for heat absorption in other systems (e.g., Lacey and Herr, 2005). We chose to measure NIR reflectance because long wavelengths have the potential to contribute to heat gain despite often being ignored because they do not contribute to the visual perception of color (Stuart-Fox et al., 2017). Measurements of reflectance were averaged for each population.

We then modeled petal color from each plant in hexagonal color space using peak photoreceptor absorbance for *Bombus impatiens,* a known pollinator of *C. americana*. Peak absorbances for this trichromatic visual system are 347, 424, and 539nm (Skorupski and Chittka, 2010). Photoreceptor absorbance is not known for another common pollinator of *C. americana*, *Megachile campanulae* (Megachilidae). We therefore used visual system data from a confamilial taxon, *Osmia rufa* (348, 436, and 572 nm; Menzel et al., 1988) to approximate petal color perceived by *M. campanulae*. For each pollinator visual system, we modeled petal color using the long-wavelength photoreceptor for achromatic receptor stimulation, standard D65 illuminance, a standard green background, hyperbolic-transformed quantum catch, and a von Kries color correction using the Pavo package in R (Maia et al., 2019). Each visual model was plotted into hexagonal color space to obtain *x* and *y* coordinates. The coordinates in color space were averaged across individuals for each population.

### Predictors of Color Variation: Climatic Data, Pollinator Data, and Population Structure

We extracted average temperature (bio10) and precipitation (bio18) of the warmest quarter from Worldclim (Hijmans et al., 2005) using the latitude and longitude of each population. We chose average summer climatic values because they are representative of the conditions experienced by flowers (see Koski and Galloway, 2018). In particular, temperature and drought are two factors shown to impose selection on anthocyanin-based petal coloration (e.g., Warren and Mackenzie, 2001; Lacey and Herr, 2005; Vaidya et al., 2018). Although other work has found duration of solar radiation influences floral color (Arista et al., 2013), preliminary analysis found no relationship for *C. americana* (results not shown).

We observed pollinator visitation to flowers in 1 m^2^ plots in each of the 24 focal populations in 2015 and 2016. In each year we observed six plots per population for 15 min each and counted the number of open flowers. We categorized visitors as small bees or large bees. The small bee category includes the common visitor *M. campanulae* and other solitary bees (see Koski et al., 2017 for detail). The bees in this category are active pollen collectors and are relatively inefficient pollinators relative to *Bombus* spp. (Koski et al., 2018). The large bee category consists almost exclusively of *Bombus* spp. Average visitation rate for each population across two years for each category was calculated as visits per flower per hour. One population was inaccessible in 2016, so only had one year of pollinator visitation data (Arkansas 56). Visitation rate did not differ between years (Koski et al., 2017).

Previous phylogenomic work based on RAD-Sequencing of 24 populations found populations of *C. americana* west of the Appalachians (i.e., those in the current study) originated from a glacial refugium in Southeastern Kentucky. The geographic distance from this refugium predicts population-level genetic diversity and both genomic and ecological genetic estimates of genetic drift (Koski et al., 2019), strongly supporting post-glacial expansion from this area. Thus, populations are genetically-structured with increasing distance from the refugium (Koski et al., 2019; Prior et al. in review). We used the linear geographic distance from the refugium as a proxy of population structure established through post-glacial migration (Koski et al., 2019).

### Statistical Analyses

To determine whether mean petal reflectance, blue reflectance, and NIR reflectance displayed clinal geographic variation across the range of *C. americana*, we modeled the population average of each metric as a function of latitude and longitude with multiple linear regressions. We then asked whether climatic, pollinator, or genetic structure contributed to any geographic variation. We modeled each reflectance parameter as a function of two climatic variables (summer temperature, summer precipitation), small bee visitation rate, *Bombus* visitation rate, and the distance from the glacial refugium using multiple linear regression. Pollinator visitation rates were log + 0.1 transformed to improve normality.

For petal color modeled in hexagonal pollinator visual space, the average population-level x- and y-coordinates were correlated (*Bombus* model, r=0.64, *P* < 0.001; *Osmia* model, r=0.66, *P* < 0.001). Therefore, we modeled the location in hexagonal visual space (*x* and *y*) using MANOVA. All MANOVA models were the same as those used to model petal reflectance measures. All statistical analyses were performed in R (R Core Team, 2020).

## Results

### Spatial Variation in Petal Reflectance and Color

**Figure 2:**
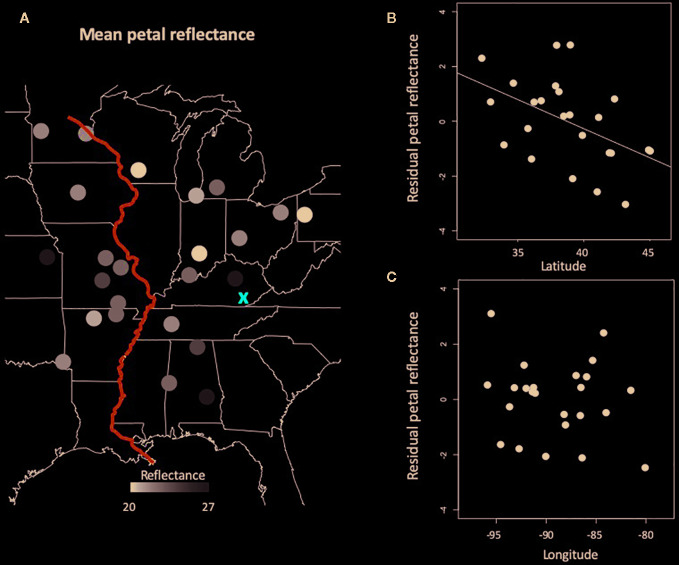
Spatial variation in mean petal reflectance among populations. **(A)** A heat map of petal reflectance across *Campanula americana*'s range with darker circles indicating less reflective petals. The red ‘X' is the most likely region of the Pleistocene glacial refugium of *C. americana*, and the blue line is the Mississippi River. **(B)** The direct effect of latitude on petal reflectance after accounting for longitudinal variation. **(C)** The direct effect of longitude on petal reflectance after accounting for latitudinal variation.

Together, latitude and longitude explained 24% of the variation in mean petal reflectance across 24 populations of *C. americana* (F_2,21 =_ 3.35, *P*=0.054, *R* ^2 =^ 0.24), but only the effect of latitude was significant (**Table 1**). Petals in more northerly populations displayed lower average reflectance from 300 to 850 nm (**Figure 2**). That is, more northern populations had darker petals. Similar patterns were observed for peak blue and NIR reflectance (**Table 1**).

**Table 1:** Spatial variation in petal reflectance attributes and pollinator-perceived color for 24 populations of *Campanula americana*.

	Mean Reflectance			Blue Reflectance			NIR Reflectance			Location in Hexagonal Color Space *Bombus impatiens*		
	Estimate	t	*P*	Estimate	t	*P*	Estimate	t	*P*	Pillai's Trace	F_2,20_	*P*
**Intercept**	27.251	4.024	0.001	34.084	3.987	0.001	35.810	3.194	0.004	–	–	–
**Latitude**	**-0.213**	**-2.514**	**0.020**	**-0.245**	**-2.295**	**0.032**	**-0.312**	**-2.225**	**0.037**	0.169	2.030	0.157
**Longitude**	-0.050	-0.736	0.470	-0.065	-0.758	0.457	-0.185	-1.637	0.116	**0.531**	**11.330**	**<0.001**
	*R^2^ = 0.24; F_2,21_ = 3.35; P=0.054*			*R^2^ = 0.21; F_2,21_ = 2.843; P=0.08*			*R^2^ = 0.26; F_2,21_ = 3.65; P=0.04*					

Reflectance was analyzed using multiple linear regression while the x-y coordinates of hexagonal color space for Bombus impatiens were modeled using MANOVA with Type II sum of squares. Statistics in bold font are significant at the P < 0.05 level.

**Figure 3:**
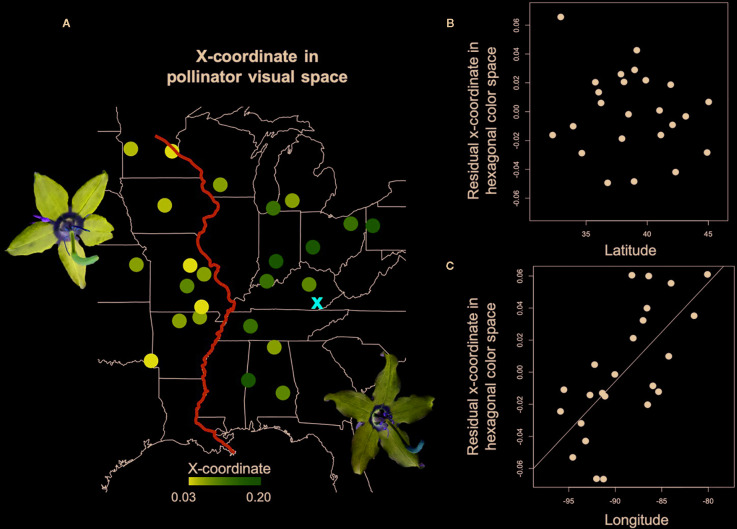
Spatial variation in petal color perceived by trichromatic *Bombus impatiens*. **(A)** A heat map of the x-coordinate in hexagonal color space for *Campanula americana*'s range with darker circles indicating lower x values. X-coordinate values are shown on the map because they are more variable among populations than y-coordinates. The red “X” is the most likely region of the Pleistocene glacial refugium of *C. americana*, and the blue line is the Mississippi River. Colors roughly represent the human-perceived color of petals. **(B)** The direct effect of latitude on the x-coordinate after accounting for longitudinal variation. **(C)** The direct effect of longitude on the x-coordinate after accounting for latitudinal variation.

Petal coloration in hexagonal visual space for *Bombus impatiens* displayed a strong longitudinal cline (**Table 1**). Populations with higher x-axis values tended to be near or within the blue-green sector of hexagonal visual space, while those with lower values were in the center of the blue sector (**Figure 1**). Thus, we visualized variation in the x-coordinate across the range (**Figure 3**). The x-coordinate in visual space decreased from west to east across the range (**Figure 3**). Results obtained using *Osmia rufa*'s visual system were similar (**Supplementary Table 1**). Thus, overall petal reflectance covaried with latitude, while petal color modeled using pollinator visual systems covaried with longitude.

### Ecological and Historical Predictors of Reflectance and Color Variation

**Figure 4:**
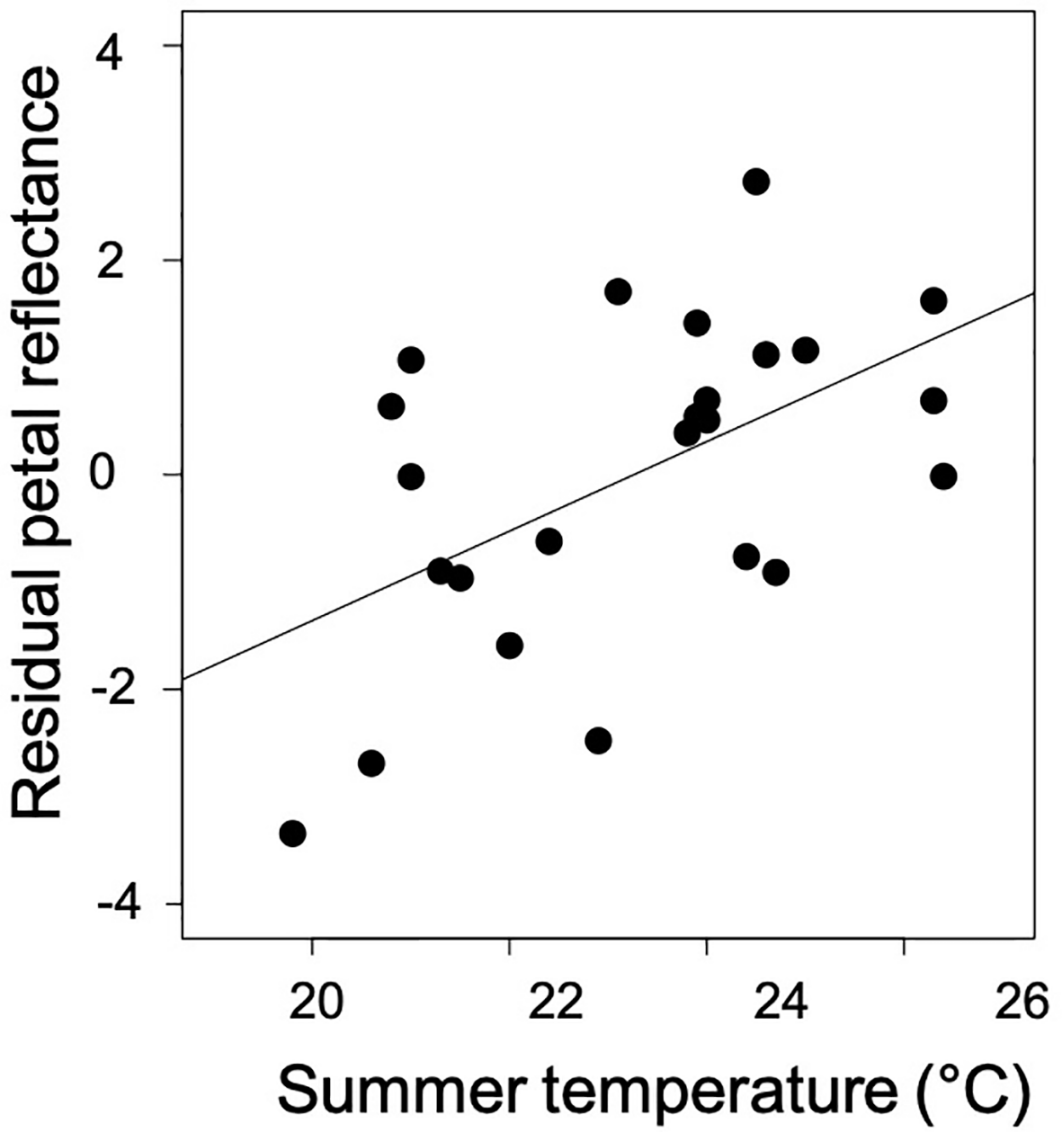
The direct effect of average temperature of the warmest quarter on mean petal reflectance after accounting for precipitation, pollinator visitation, and the post-glacial colonization (distance from glacial refugium; see **Table 2**).

In the model testing the effects of climatic variables, the pollinator community, and the distance from the glacial refugium on petal coloration, only temperature predicted petal reflectance (**Table 2**; **Figure 4**). Temperature was positively associated with overall, blue, and NIR reflectance (**Table 2**).

**Table 2:** Effects of temperature, precipitation, pollinator visitation, and post-glacial colonization on petal reflectance attributes and pollinator-perceived color for 24 populations of *Campanula americana*.

	Mean Reflectance			Blue Reflectance			NIR Reflectance			Location in Hexagonal Color Space, *Bombus impatiens*		
	Estimate	t	*P*	Estimate	t	*P*	Estimate	t	*P*	Pillai's Trace	F_2,17_	*P*
**Intercept**	8.685	1.568	0.134	12.625	1.802	0.088	18.065	1.920	0.071	–	–	–
**Summer Temp.**	**0.452**	**2.854**	**0.011**	**0.519**	**2.595**	**0.018**	**0.757**	**2.814**	**0.012**	0.135	1.322	0.293
**Summer Precipitation**	0.014	1.037	0.313	0.018	1.109	0.282	0.007	0.328	0.747	0.166	1.693	0.214
**Small bee visitation**	0.260	0.697	0.495	0.269	0.570	0.576	0.820	1.296	0.211	0.276	3.246	0.064
***Bombus* visitation**	-0.145	-0.528	0.604	-0.068	-0.197	0.846	-0.248	-0.533	0.601	0.078	0.718	0.502
**Km from Refugium**	0.000	-0.459	0.652	0.000	-0.360	0.723	0.001	0.505	0.620	**0.603**	**12.936**	**<0.0001**
	*R^2^ = 0.38; F_5,18_ = 2.17; P=0.104*			*R^2^ = 0.35; F_5,18_ = 1.94; P=.138*			*R^2^ = 0.36; F_5,18_ = 1.99; P=0.128*					

Reflectance was analyzed using multiple linear regression while the x-y coordinates of hexagonal color space for Bombus impatiens were modeled using MANOVA with Type II sum of squares. Bold values indicate significance at P < 0.05.

**Figure 5:**
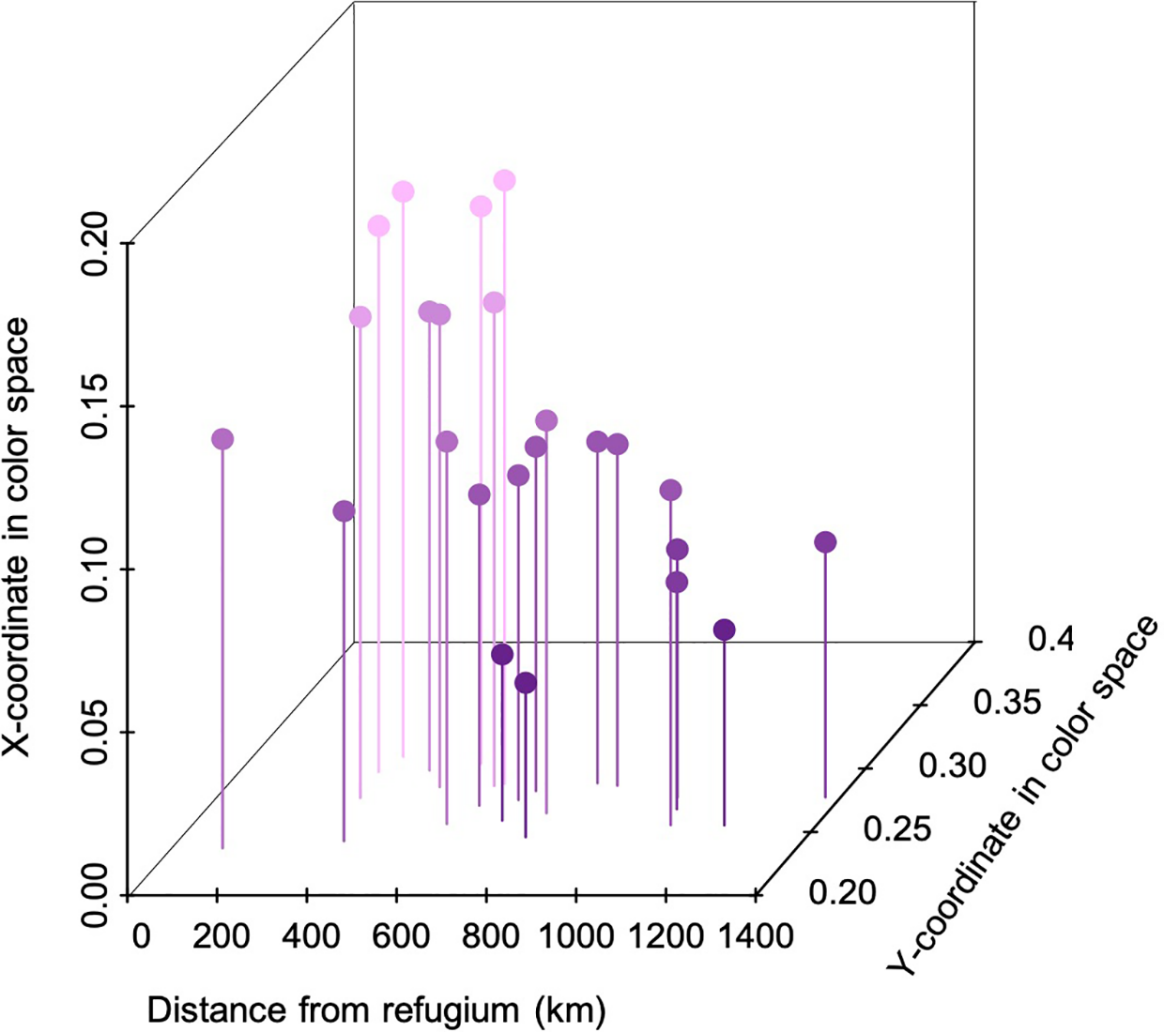
The relationship between petal color perceived by trichromatic pollinator *Bombus impatiens,* and the distance from the glacial refugium. X and y- coordinates corresponds to **Figure 1B** and the location of the glacial refugium is denoted in **Figures 2** and **3**.

When petal reflectance was modeled with trichromatic pollinator visual systems, there was a strong effect of the distance from the glacial refugium on the location of petal color in pollinator visual space (**Table 2**). With increasing distance from the refugium, populations had smaller x- and y-coordinates in hexagonal color space of *Bombus impatiens* (**Figure 5**). Additionally, there was a marginally significant effect of small bee visitation on pollinator-perceived color (p=0.06; **Table 2**). Specifically, flowers had smaller x and y coordinates in populations with higher small bee visitation (**Supplementary Figure 1**). Results were consistent when the visual system of *Osmia rufa* was used to model color (**Supplementary Table 2**).

## Discussion

Range-wide geographic patterns of petal coloration in *C. americana* appear to be largely shaped by the thermal environment and genetic structuring imposed by post-glacial range expansion. Petal reflectance, which has been shown to function in floral thermoregulation in other systems, displayed a latitudinal pattern with petals being less reflective in colder environments. However, petal color modeled with trichromatic pollinator visual systems displayed a strong longitudinal pattern that is largely consistent with flower color evolving in concert with post-glacial range expansion. The role of more contemporary pollinator environments in driving color variation is less important than the role of historical expansion, a finding that is similar to the geographic structuring of other floral traits in *C. americana* (e.g., selfing ability; Koski et al., 2017). Thus, this work underscores the importance of the combination of neutral and adaptive evolutionary forces when examining the evolution of flower color.

### Temperature as a Driver of Latitudinal Variation in Petal Reflectance

As predicted, petals were less reflective across the UV to NIR spectrum in more northern populations that experience cooler conditions during flowering. The latitudinal pattern was only associated with temperature. That is, there was no detectable influence of the pollinator community, post-glacial expansion or precipitation on petal reflectance in our models. Interestingly, previous phylogenomic work in *C. americana* suggests the potential for distinct genetic lineages east and west of the Mississippi River, and if the dataset is split by this barrier petal reflectance in both groups is elevated in populations that experience higher temperatures (Eastern group, r=0.60, *P*=0.037; Western group, r=0.53, *P*=0.078). These correlations bolster support for temperature contributing to the evolution of petal reflectance in *C. americana*. In *Plantago lanceolata*, cold temperatures result in increased floral anthocyanin production in inflorescences, increasing absorption, especially in the NIR range (Stiles et al., 2007). Increased light absorption warms reproductive structures while increased reflectance can cool reproductive structures or simply minimize solar heat gain (Lacey and Herr, 2005). Our results join Lacey et al. (2010) in showing a strong latitudinal pattern of reduced reflectance in cooler, northern populations. Since *C. americana* were grown in a common environment, the reflectance measurements capture fixed genetic differences among populations.

The pattern of reduced petal reflectance in cooler populations and elevated reflectance in warmer populations could be driven by a number of potential mechanisms. First, floral warming and cooling could benefit pollen and ovules in cold and warm conditions, respectively. Second, floral warming and cooling could increase pollinator visitation in cool and warm conditions, respectively. Finally, the latitudinal cline in petal reflectance could be due to plant-wide upregulation of the anthocyanin biosynthetic pathway under cooler temperatures, in which case there may be no adaptive role of variation in petal reflectance.

The viability of *C. americana* pollen is reduced by high heat stress, and lighter pollen color morphs (white/tan) incur more damage than darker purple pollen morphs (Koski and Galloway, 2018). Thus, increased petal reflectance, which has the potential to cool flowers (van der Kooi et al., 2019), should be favored in warmer southern populations that experience heat stress (Koski and Galloway, 2018). In contrast, an experimental cold temperature treatment (13°C) did not affect pollen viability in *C. americana,* therefore the effect of petal reflectance on warming in northern populations is unlikely driven by selection to increase pollen performance. Low temperatures have the ability to reduce ovule viability in other systems (e.g., Thakur et al., 2010). Thus, in cooler northern *C. americana* populations, increased absorption may warm flowers and increase ovule performance. However, given the inferior ovary of *C. americana* flowers, petal reflectance may have a minimal effect on ovule temperature.

Floral warming in the north and cooling in the south through altered petal reflectance could be the result of adaptation for increasing pollinator visitation. Warmer flowers experience elevated pollinator visitation in cold environments (Norgate et al., 2010). Conversely, pollinators have been shown to overheat in warmer flowers during extreme heat (Corbet and Huang, 2016), and choose cooler flowers at high temperatures (Shrestha et al., 2018). Small pollinators have been observed basking in flowers of *C. americana* (L. Galloway, pers. obs.), thus petal reflectance may affect pollinator thermal preferences with consequent impacts on plant reproductive success.

Finally, cold temperatures typically lead to upregulation of the anthocyanin biosynthetic pathway throughout the plant (Chalker-Scott, 1999) which could result in increased light absorption by petals in northern populations. Positive correlations in anthocyanin concentrations between petal and vegetative tissues can be strong (e.g., del Valle et al., 2015). In a mustard polymorphic for flower color, the frequency of the non-pigmented morph declined in the coldest populations, suggesting that anthocyanins are crucial for plant performance under cold stress (Dick et al., 2011). We posit that in *C. americana* darker petals have higher concentrations of anthocyanins and the latitudinal cline in petal reflectance could be driven by plant-wide adaptive responses to temperature across the wide latitudinal gradient.

### Historical Colonization, Not Pollinators, as a Driver of Petal Color Variation

While mean petal reflectance displayed a latitudinal pattern, petal coloration modeled using the visual system of two separate trichromatic bee pollinators displayed a strong longitudinal pattern. The distance from the glacial refugium had the strongest effect on pollinator-perceived petal color, but there was also a modest and near-significant influence of small bee visitation rate. Historical migration of *C. americana* from the southern Appalachian plateau following glacial recession has resulted in genetic clusters of populations east and west of the Mississippi River (Koski et al., 2019; Prior et al. in revision). Geographic differences in flower color among populations are consistent with a Mississippi River split (**Figure 3**). Thus, we conclude that population structure driven by post-glacial expansion has been instrumental in shaping color variation in *C. americana*. To our knowledge, this is the first study to support that post-glacial migration contributes to geographic variation in a floral trait traditionally considered to influence pollinator attraction.

The link between petal color and post-glacial migration suggests that one of two evolutionary processes contributed to longitudinal petal color variation. First, neutral genetic drift occurred with range expansion, driving color evolution. Alternatively, novel selective pressures experienced in new habitats during colonization acted either directly on petal color or on traits correlated with petal color. Novel selection pressures may have included strong pollen and/or pollinator limitation or unique abiotic conditions. Alternatively, correlations between petal color and traits that are likely under strong selection during range expansion (like autonomous-selfing) may contribute to the structuring of petal color through range expansion. Among populations there is a correlation between the X-coordinate in *Bombus* color visual space and autonomous fruit set (r = -0.44, *P*= 0.03), though whether there is a functional link between selfing ability and coloration is unknown. For instance, the trait correlation could reflect independent evolution of each trait during range expansion, or a genetic correlation that did not evolve adaptively. The effects of neutral evolution during expansion and historical adaptation on the geographic pattern in flower color are difficult to disentangle. However, we predict that neutral genetic processes shaped the geographic pattern in petal color among *C. americana* populations, with any evolution due to contemporary pollinator or abiotic conditions occurring within that genetic structure, and hence resulting in a more modest impact on the phenotype.

We posit that alterations to petal color in *C. americana* are likely achieved through genetic modification to regulatory or structural anthocyanin biosynthetic pathway genes, resulting in a change in the ratios of colored anthocyanin compounds (i.e., copigmentation; Asen et al., 1972). While the pigment biochemistry of petals is not known, variation in pollen color in *C. americana* is driven largely by changes in concentrations of the anthocyanins cyanidin and peonidin (Koski et al., 2019). Populations east of the Mississippi River had petals closer to the “blue-green” sector of color space while those west of the Mississippi River had petals near the center of the “blue” sector of color space (**Figure 1**). This pattern was driven in part by a modest shift in the blue peak towards the pollinators mid-wavelength (green) photoreceptor in eastern populations. Populations with an average peak of reflectance near 440nm had higher x- and y- coordinates than populations with a peak near 435 nm (blue peak wavelength and x-coordinate correlation *r*=0.87, P < 0.0001; blue peak wavelength and y-coordinate correlation, *r*=0.41, P=0.05*).*Thus, the geographic pattern in pollinator-perceived color is due to a modest (5 nm) peak shift in reflectance in the blue range potentially driven by changes in copigmentation (Asen et al., 1972) that occurred in concert with range expansion.

Interestingly, pollen color in *C. americana* displays a longitudinal cline that has been largely attributed to selection by the thermal environment (Koski and Galloway, 2018). Specifically, pollen was deeper purple in western populations that experience higher summer temperatures and deeper purple pollen is more heat tolerant. However, Koski and Galloway (2018) scored pollen color by eye which does not incorporate reflectance of light outside of the wavelengths of human visual perception (~400-700 nm) nor does it model pollinator perception of color. Thus, the color metrics of petals from this study cannot be easily compared to the pollen color scored in Koski and Galloway (2018). Regardless, given the strong longitudinal cline in pollen color, there is the potential that historical colonization has played a role in structuring pollen color as well as petal color despite a lack of genetic correlation between the two (Koski et al., 2020).

## Conclusions

Different components of petal reflectance appear to be shaped by different evolutionary forces in *C. americana*. Geographic variation in the intensity of petal reflectance is governed by temperature while pollinator-perceived color is governed by population structure established through post-glacial migration. Because these results in *C. americana* are correlational, additional tests of whether petal reflectance shows similar patterns across temperature gradients and examinations of how historical migration has influenced color evolution in more taxa will be important for understanding drivers of large scale biogeographic structuring in flower color.

## Data Availability Statement

All datasets presented in this study are included in the article/**Supplementary Material**.

## Author Contributions

MK and LG conceptualized the study. MK collected and analyzed the data. LG provided feedback on data analyses and interpretation. MK wrote the manuscript with input from LG.

## Conflict of Interest

The authors declare that the research was conducted in the absence of any commercial or financial relationships that could be construed as a potential conflict of interest.
