# Supplementary material for: Geographic Variation in Floral Color and Reflectance Correlates With Temperature and Colonization History
Source: Front Plant Sci. 2020 Jun 30;11:991. doi: 10.3389/fpls.2020.00991 (PMC7340105; doi:10.3389/fpls.2020.00991)

Supplementary Materials for Koski & Galloway 2020, "Temperature and historical colonization shape geographic variation in petal reflectance and coloration"

Supplementary Table 1: MANOVA results testing the effect of latitude and longitude on petal color perceived by *Osmia rufa*.

Effect	Pillai's Trace	$F_{2,20}$	*P*
**Latitude**	0.186	2.281	0.128
**Longitude**	**0.536**	**11.548**	**<0.001**

Supplementary Table 2: Results from MANOVA testing the effect of temperature, precipitation, pollinator visitation, and post-glacial colonization on petal color perceived by *Osmia rufa*.

	Pillai's Trace	$F_{2,17}$	*P*
**Summer Temp.**	0.148	1.477	0.256
**Summer Precipitation**	0.142	1.406	0.272
**Small bee visitation**	0.263	3.038	0.074
***Bombus* visitation**	0.060	0.540	0.593
**Km from Refugium**	**0.600**	**12.760**	**<0.0001**

Supplementary Figure 1: The relationship between ineffective small bee visitation rate (*Megachile campanulae* + various other small solitary bee species) and flower color in hexagonal color space of *Bombus impatiens*.

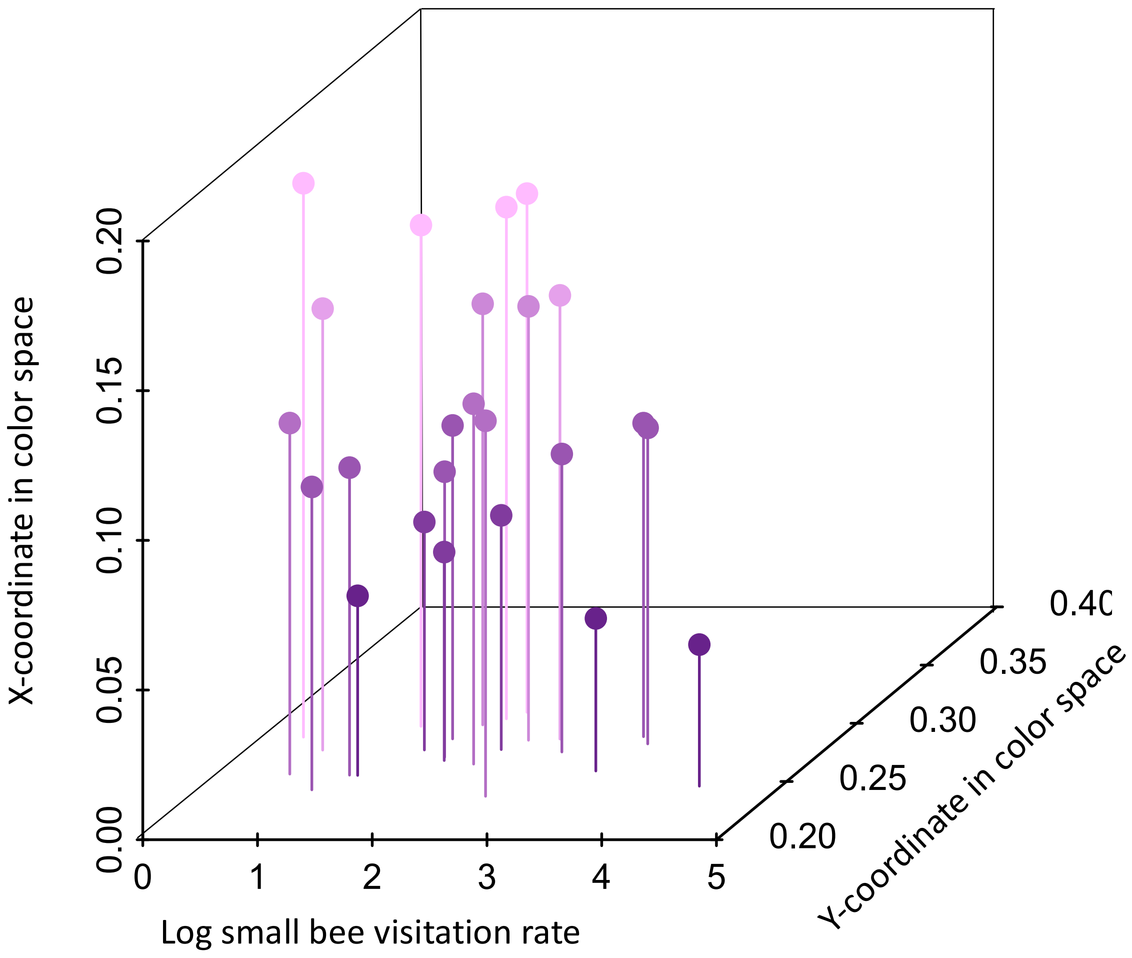

Supplement: Supplementary file 1 [file DataSheet_1.pdf]
